# North African hybrid sparrows (*Passer domesticus*,* P. hispaniolensis*) back from oblivion – ecological segregation and asymmetric mitochondrial introgression between parental species

**DOI:** 10.1002/ece3.2274

**Published:** 2016-06-28

**Authors:** Abdelkrim Ait Belkacem, Oliver Gast, Heiko Stuckas, David Canal, Mario LoValvo, Gabriele Giacalone, Martin Päckert

**Affiliations:** ^1^ Faculty of Sciences of nature and lifes Department of AgropastoralismUniversity of DjelfaBP. 311717000DjelfaAlgeria; ^2^ Senckenberg Naturhistorische SammlungenKönigsbrücker Landstraße 159D‐01109DresdenGermany; ^3^ Institute of Vertebrate BiologyCzech Academy of SciencesExternal Research Facility StudenecStudenec 122675 02KoněšínCzech Republic; ^4^ Department of Evolutionary EcologyEstación Biológica de Doñana – CSICAvda. Américo Vespucio s/n41092SevilleSpain; ^5^ Dipartimento di Scienze e Tecnologie BiologicheChimiche e FarmaceuticheVia Archirafi 18I‐90123PalermoItaly; ^6^ Cooperativa SileneVia Dondes Regio, 8/a90127PalermoItaly

**Keywords:** Agricultural landscape mosaic, Algeria, breeding phenology, NADH dehydrogenase, nest site choice

## Abstract

A stabilized hybrid form of the house sparrow (*Passer domesticus*) and the Spanish sparrow (*P. hispaniolensis*) is known as *Passer italiae* from the Italian Peninsula and a few Mediterranean islands. The growing attention for the Italian hybrid sparrow and increasing knowledge on its biology and genetic constitution greatly contrast the complete lack of knowledge of the long‐known phenotypical hybrid sparrow populations from North Africa. Our study provides new data on the breeding biology and variation of mitochondrial DNA in three Algerian populations of house sparrows, Spanish sparrows, and phenotypical hybrids. In two field seasons, the two species occupied different breeding habitats: Spanish sparrows were only found in rural areas outside the cities and bred in open‐cup nests built in large jujube bushes. In contrast, house sparrows bred only in the town centers and occupied nesting holes in walls of buildings. Phenotypical hybrids were always associated with house sparrow populations. House sparrows and phenotypical hybrids started breeding mid of March, and most pairs had three successive clutches, whereas Spanish sparrows started breeding almost one month later and had only two successive clutches. Mitochondrial introgression is strongly asymmetric because about 75% of the rural Spanish sparrow population carried house sparrow haplotypes. In contrast, populations of the Italian hybrid form, *P. italiae*, were genetically least diverse among all study populations and showed a near‐fixation of house sparrow haplotypes that elsewhere were extremely rare or that were even unique for the Italian Peninsula. Such differences between mitochondrial gene pools of Italian and North African hybrid sparrow populations provide first evidence that different demographic histories have shaped the extant genetic diversity observed on both continents.

## Introduction

Hybridization has been considered as the driving force of speciation in several groups of organisms (review in: Abbot et al. 2013). In birds, however, hybridization processes were most intensely studied in the context of a breakdown of reproductive barriers in secondary contact rather than with respect to the emergence of truly stabilized hybrid species. A great number of narrow‐range avian hybrid zones have been circumscribed in the Palearctic, but in most areas, distribution range of hybrids is limited to rather narrow zones of secondary contact in coexistence with both parental species (Haffer 1989; Aliabadian et al. 2005). In contrast, genetic evidence of hybrid origin of a bird species is rare, being reliably documented for a handful of taxa only, for example, for the Haiwaiian duck (*Anas wyvilliana*; Lavretsky et al. 2015), the imperial pheasant (*Lophura imperialis*; Hennache et al. 2003), Audubon's warbler (*Dendroica aududoni*; Brelsford et al. 2011), and for the Italian sparrow (*Passer italiae*), a stabilized hybrid form from the Italian Peninsula. The Italian sparrow occupies a wide distribution range in absence of either of its parental species: the house sparrow (*P. domesticus*) and the Spanish sparrow (*P. hispaniolensis*; Elgvin et al. 2011; Hermansen et al. 2011, 2014; Trier et al. 2014). This peculiar sparrow has obtained the straightforward recognition from the scientific community worldwide as a homoploid hybrid species (Gill 2014; Arnold 2016; : p. 17–18), and its proposed species rank was accordingly accepted by many taxonomic authorities (e.g., Clements et al. 2000; Dickinson and Christidis 2014). The limited geographic distribution range of the Italian sparrow is a sideline aspect rendering further credibility to the suggested species status of the Italian sparrow. According to recent genetic studies, the hybrid Italian sparrow would be distributed only throughout the entire Italian peninsula and in a few insular populations on Sicily, Sardinia (see maps in Elgvin et al. 2011; Hermansen et al. 2011; Trier et al. 2014), Malta, and Crete (Clement 1999). Despite the existence of wide areas of sympatry among the two parental species in Europe and Central Asia, hybrid phenotypes have been rarely documented in Eurasia north of the Alps. Previous work on the Iberian Peninsula reported rare hybrid individuals from areas of local contact (Alonso 1985), but there is recent evidence for genetic incompatibilities between the two parental species from a mixed Spanish population (Hermansen et al. 2014).

**Figure 1:**
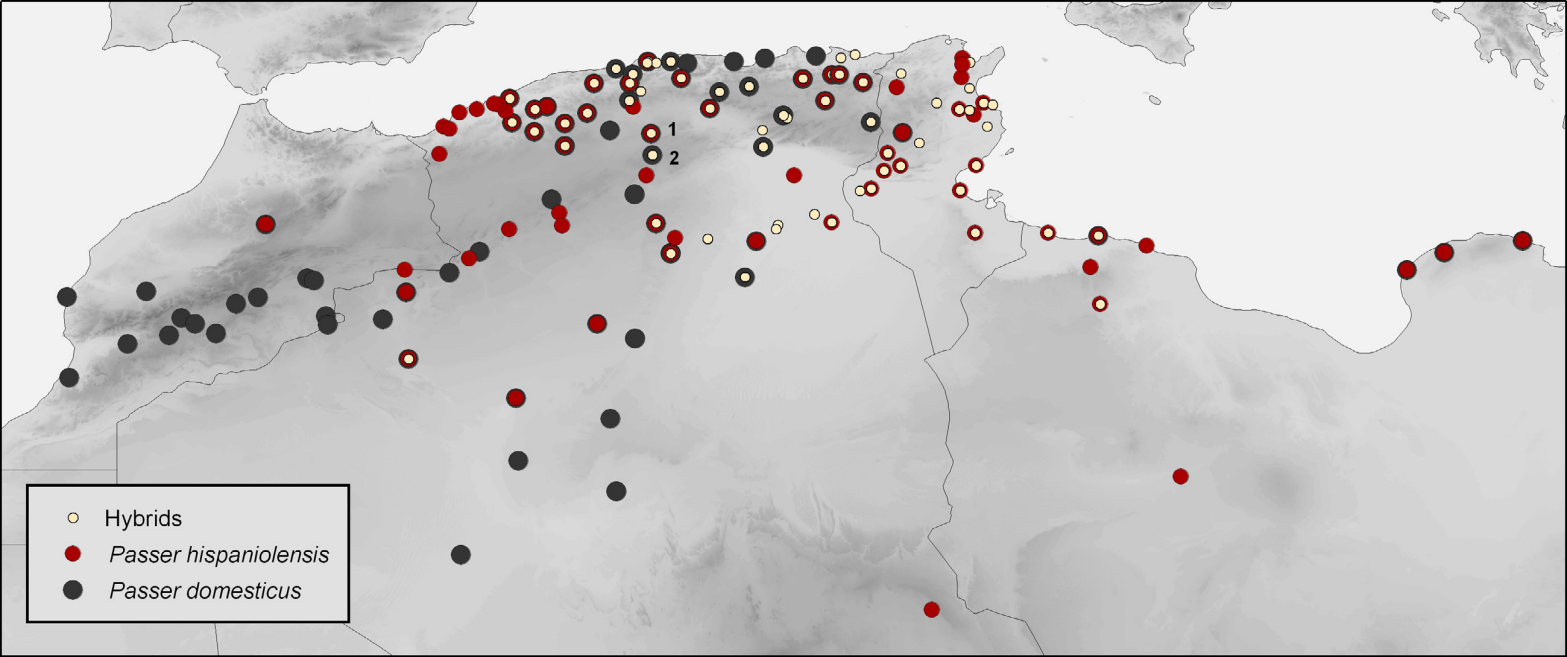
Distribution of the house sparrow (*P. domesticus*, dark gray), Spanish sparrow (*P. hispaniolensis*, dark red‐brown), and hybrid phenotypes (light beige) in North Africa; different symbol sizes were chosen to show local co‐occurrence of the three forms; main sources for occurrence data: Meise (1936), Johnston (1969), Summers‐Smith and Vernon (1972), and Metzmacher (1986); our study sites in Algeria: (1) Hassi‐El Euch; (2) Djelfa.

The situation on the Eurasian continent is greatly contrasted by the distribution pattern of the two – respectively, three – sparrow species south of the Mediterranean, on the African continent. In fact, a great number of ornithological papers have documented sparrow populations that are phenotypically intermediate between the house sparrow and the Spanish sparrow throughout a vast region in North Africa (review in Töpfer 2006: p. 120–121). A first cartographic documentation of putative hybrid sparrow populations in North Africa by Meise (1936) was later complemented and improved by contributions from other authors (Johnston 1969; Summer‐Smith and Vernon 1972; Metzmacher 1986; Haffer and Hudde 1997). Recent avifaunistic surveys demonstrated that North African “hybrid sparrows” are “locally highly abundant” with local records from Algeria accounting “more than one‐third of the total number of individuals of all species inventoried” (Guezoul et al. 2010; Guezoul et al. 2011; Bendjoudi et al. 2013). Thus, the spatial distribution of house and Spanish sparrows in North Africa resembles a mosaic of allopatric and sympatric populations with more than 50 occurrence records of the intermediate phenotype (Fig. 1). Summer‐Smith and Vernon (1972) described the situation in North Africa as a “spatially diffuse hybrid zone extending from eastern Algeria through Tunisia into eastern Tripolitania” (illustration in Summers‐Smith 1988, fig. 44). However, to date, reliable data on life history traits and the genetic constitution of these North African sparrow populations are still missing.

Here, we provide field data on the breeding biology (habitat preferences, phenology, and reproductive success) of *P. domesticus*,* P. hispaniolensis,* and their putative hybrids from two breeding seasons on two study sites in Algeria. Throughout their sympatric European range, breeding phenology and nest site preferences of house sparrows and Spanish sparrows are partly diverged (Alonso 1986; Murgui 2011; Tu 2012) and our field study aims to determine whether such ecological segregation occurs in North African populations. Field data are backed by morphometric comparisons among the three locally coexisting phenotypes and by genetic analysis of mitochondrial DNA. As is known, the house sparrow mitogenome has almost completely introgressed into the Italian hybrid form, *P. italiae* (about 98% of study individuals of Hermansen et al. 2011) and we expect to confirm such a fixation of *P. domesticus* haplotypes for North African hybrids, too. Considering the effective reproductive barriers between House sparrows, Spanish sparrows on the Iberian Peninsula (Hermansen et al. 2014), and between the Italian hybrid form and the parental species throughout the Italian Peninsula (Trier et al. 2014), we expect a rather limited degree of mitochondrial introgression between *P. domesticus* and *P. hispaniolensis* in Algeria.

## Material and Methods

### Study area

Fieldwork and nest surveys were carried out in the years 2011 and 2012 at two locations: Hassi El‐Euch (35°9′ North; 3°14′ East, 910 m. a. s. l) and Djelfa (34°40′ North; 3°15′ East, 1138 m.a.s.l), in the region of Zehrez, located at about 275 km in the southeast of Algiers (Fig. 1). The region is semi‐arid with relatively cool winters and precipitations ranging from 217 to 337 mm (Belkacem et al. 2012). The Institut de Technologie Moyen Agricole Spécialisée (ITMAS) was our study site at Djelfa. The institute, located in an urban environment, has an area of ca. 16 ha and consists of buildings and houses surrounded by agricultural study lots for wheat production and cattle farming, some of them bordered by small stands of Aleppo pine (*Pinus halepensis*). In Hassi‐El Euch, we studied two sparrow populations: the urban study site was located at the local school and the surrounding quarters that were bordered by fallow land in the North, whereas the rural study site consisted of large agricultural fields of wheat covering about 1450‐ha extent. The abundance of jujube bushes – the preferred breeding habitat of Spanish sparrows – in the area was rather low (2% of the total plot; see Belkacem et al. 2012).

Fieldwork started with the onset of the breeding period of house sparrows in mid‐March (15th March in both study periods). Nests were regularly checked to ascertain laying date, clutch size, hatching date, and number of fledglings of first, second, and third clutches.

We used general and generalized linear models (GLM) to explore for differences among populations in laying dates (Gaussian distribution), clutch sizes (Poisson distribution), and reproductive success (young fledged in relation to clutch size; binomial distribution). If differences were significant, post hoc Tukey's test was applied to test for paired differences between the three study populations. In these models, laying date was included as a predictor because may be an important determinant of reproductive success in birds (Newton 2008). Further, year was included as a random effect to account for between‐year variability in environmental conditions influencing reproductive success. Selection of the minimum adequate models was conducted by sequentially dropping nonsignificant terms from fully saturated models (containing all main effects and interactions) in a hierarchical way, starting with the least significant order terms. We systematically performed model diagnostics statistics while modeling to avoid misleading conclusions based on statistical artifacts. As we performed multiple tests using the phenologic data set, we applied the Benjamini–Yekutieli correction for multiple tests (which is more appropriate than other methods of correction for multiple comparison tests; Narum 2006).

### Morphology

Phenotypical assignment of individual birds to either of the two sparrow species was based on diagnostic plumage traits (Fig. S1). These refer to the color pattern of the crown, the nape, the cheek and the back, the extent of black chest patch, and the presence and absence of black‐streaked pattern on the flanks (following Summers‐Smith 1988; Cramp and Perrins 1994). Phenotypic hybrid individuals were identified according to their intermediate plumage characters, particularly with respect to crown color, cheek color, and black spots on the flanks and underparts (Fig. S1). To take into account the considerable variation among hybrid phenotypes, different methods of quantifying a hybrid index have been established (Meise 1936; Johnston 1969; Lo Valvo and Lo Verde 1987) and applied for the classification of hybrid phenotypes across an alpine contact zone between *P. domesticus* and the hybrid form *P. italiae* (Hermansen et al. 2011). Because all these hybrid indices were based on estimated numerical values that had never been quantified or measured (see Töpfer 2006), they are all highly subjective and not directly comparable. Therefore, for our analyses of Algerian populations, we classified all intermediate phenotypes as phenotypical hybrids opposed to the 100% parental *domesticus* and *hispaniolensis* phenotypes (compare Fig. S1). In the context of the present study, the use of this binomial method may have led to a slight underestimation of phenotypic hybrid numbers. Thus, our results likely represent a conservative estimate of the actual situation in Algerian populations.

Although distinction of the two sparrow species is easy in males, females of both species are almost indistinguishable from a distance (Cramp and Perrins 1994). Generally, females of both species are very much alike, but in comparison, Spanish sparrows have somewhat paler crown and face, distinct dusky furrows at the flanks, a whiter belly and sharper streaks on mantle (Cramp and Perrins 1994; Haffer and Hudde 1997). However, none of these characters is really diagnostic and, therefore, in cases of doubt, we finally classified females according to the phenotype of the males they were paired with (in accordance with Hermansen et al. (2011), who classified females according to “geographical location and the corresponding male phenotype”). This classification is naturally prone to error because it *a priori* neglects any kind of female mating preference, however, for the phenotypical classification of females there is barely an alternative.

We used of 245 individuals for morphometric analysis. Birds were weighed (to the nearest 0.1 g) and measured for body length (to the nearest 0.5 mm), from the tip of the bill to the tip of the longest tail feather. Wing span was measured as the distance between wing tips with both wings stretched, tarsus length as the distance between the tarsal joint and the metatarsal joint, and beak length was measured from the skull to the tip of the bill (bill‐to‐skull; Eck et al. 2011). To analyze differences between species and sexes, we ran GLM for each trait of interest (i.e., body mass, wing span, tarsus, and beak length) including the interaction between species and sex as a predictor. If differences were significant, post hoc Tukey's test was applied to test for paired differences in the traits between species (*P. domesticus*,* P. hispaniolensis,* and hybrids). In addition, we also run a model using the scores from a principal component analysis (PCA; using both the covariance and the correlation matrix approach) based on the morphometric measures detailed above. PCA was calculated including and excluding mass, as this trait is not structural and may largely vary along the day and breeding stage. Results using both sets of variables were qualitatively similar, and therefore, we only show here data from PCAs excluding mass to avoid unnecessary repetition. Selection of the minimum adequate models was as described above.

Statistical analyses on the breeding biology and morphometry of sparrows were implemented in R 3.1.2 (R Development Core Team 2015) and SPSS 14.0.

### Genetic analysis

We used a total of 185 samples belonging to eleven populations of house sparrows and Spanish sparrows. Samples are stored at the facilities of the Senckenberg Natural History Collection Dresden in 95% ethanol or thymol tissue‐buffer at −85°C until further use.

In Algeria, we collected samples from three populations in two localities: two populations constituted by house sparrows and phenotypical hybrids (Djelfa: *n* = 43, 28 *P. domesticus* + 15 hybrids; urban, Hassi El‐Euch: n = 19, 10 *P. domesticus* + 9 hybrids) and one constituted by Spanish sparrows (rural, Hassi El‐Euch: *n* = 26). Due to the crucial assignment of female phenotypes, we included only male specimens into the analysis. Algerian populations were compared (1) with populations from continental Europe and North Africa of either of the two parental species *P. hispaniolensis* (Sevilla, Spain, *n* = 22; Egypt, *n* = 7) and *P. domesticus* (Dresden, Germany, *n* = 17; Morocco, *n* = 6) and (2) with populations of *P. italiae* from Sicily and Mediterranean islands (Fraginesi, W Sicily, *n* = 10; Maletto, E Sicily, *n* = 11; Ustica island, *n* = 9; Lampedusa island, *n* = 15). Although being phenotypically rather similar to *P. hispaniolensis,* these populations from Sicily and neighboring islands are usually thought to represent the Italian stabilized hybrid form. The population from Lampedusa is mostly neglected the recent taxonomic standard literature, and when discussed, it is rather affiliated to the Spanish sparrow, *P. hispaniolensis* (Massetti 2009). However, like on other Mediterranean islands and similar to sparrows from Sicily, the birds in our study population from Lampedusa were phenotypically close to *P. hispaniolensis* but completely lacked the typical black breast and flank stripes (compare Fig. S2). Therefore, we also classified the Lampedusa population as belonging to the hybrid form *P. italiae*.

To estimate the dimension of introgression of parental mitochondrial lineages into North African hybrid populations, we chose the NADH dehydrogenase subunit 2 (ND2) as a marker gene for comparison with sequence data available from previous studies (Elgvin et al. 2011; Hermansen et al. 2011). Mitochondrial ND2 lineages of house sparrows and Spanish sparrows are strongly diverged, but local gene pools of the Italian hybrid form *P. italiae* are composed to near 100% of house sparrow haplotypes (Hermansen et al. 2011) and we would expect a similar situation in North African hybrids.

For comparison with our own sequence data set, we added 47 ND2 sequences from two further European populations studied in Hermansen et al. (2011): Norwegian *P. domesticus* from Olso (GenBank numbers: JN090466–JN090481). *P. hispaniolensis* from Sardinia (GenBank numbers: JN090483–JN090497), and *P. italiae* from Central Italy (GenBank numbers: JN90498–JN90512).

DNA was extracted from tissue samples in a standard chloroform–isoamylalcohol extraction using dodecyltrimethylammonium bromide (DTAB) that followed a modified protocol for blood samples (Gustincich et al. 1991). For amplification of the entire 1079‐bp long ND2 gene, we used a pair of external primer pairs: H6313 (5′‐ACT CTT RTT TAA GGC TTT GAA GGC‐3′) and L5216 (5′‐GGC CCA TAC CCC GRA AAT G‐3′). The polymerase chain reaction (PCR) set‐up consisted of 25 *μ*L total volume each containing 2.5 *μ*L 10× PCR buffer, 0.5 *μ*L dNTPs (10 mM), 1.0 *μ*L of each primer (10 pmol), 0.2 *μ*L *Taq* polymerase, 20–50 ng template DNA, and ddH2O to yield a final volume of 25 *μ*L. Negative controls containing no DNA template were run with each PCR to rule out an effect of possible contamination. We empirically determined the optimal annealing temperature of 57°C for our PCR primer pair in a gradient PCR. Except for that little modification, the PCR protocol followed that of Hermansen et al. (2011).

Because we received suboptimal sequencing results when using one of the external primers H6313 or L5216 for a considerable amount of samples, we designed two internal sequencing primers using OLIGOANALYZER 1.03 Software (Owczarzy et al. 2008) and a preliminary ND2 alignment including sequences from both species: forward primer PasserND2_seqintF, 5′‐ACC ATC ACT AAA TCC CAC ACT C‐3′; reverse primer PasserND2_seqintR 5′‐TAA GGT GAG GAA GAC TGT TGA G‐3′). We used the BigDye^®^ Terminator Cycle Sequencing Kit (Applied Biosystems Inc., Darmstadt, Germany) to sequence the ND2 PCR products. Sequencing reactions (*V*_f_ = 10 *μ*L) consisted of 50–100 ng DNA template per 1000 bp (in most cases 1 *μ*L PCR product), 1 *μ*L Primer (5 pmol), 0.5 *μ*L BigDye, 2.25 *μ*L 5× sequencing buffer, and 5.25 *μ*L ddH2O. Sequencing PCR products were purified using Sephadex^™^ purification. Sequencing analysis of PCR products was performed on an ABI3730xl capillary sequencer (Applied Biosystems).

All 145 ND2 sequences (own samples and GenBank sequences, 717 bp length) were manually aligned using MEGA 5.1 (Tamura et al. 2011). Variable and ambiguous sites were visually checked for accuracy and validated by examining the raw data electropherogram output file. Nucleotide sequences were translated into protein sequences with MEGA 5.1 in order to control for stop codons and thus to exclude numt (nuclear mitochondrial DNA) sequences as a potential source of error. For genetic analyses, the alignments were cut at the end of both strands and sequences with missing data or ambiguous sites were eliminated. ND2 sequence data set was deposited at GenBank under accession numbers KX370619‐KX370815.

We reconstructed a minimum‐spanning haplotype network using TCS 1.2.1, phylogenetic network estimation using statistical parsimony (Clement et al. 2000), and calculated genetic diversity indices with DnaSP 5.10 (Librado and Rozas 2009). DnaSP was also used to infer the mismatch distributions under a model of population growth and decline (Rogers and Harpending 1992; Rogers 1995). Expected distributions were calculated by a priori estimating theta initial and tau from the original sequence data for each population separately. To test for deviations from neutrality, we calculated Tajima's D (Tajima, 1989) and performed the McDonald–Kreitman test (McDonald and Kreitman 1991) with DnaSP V. 5.10 (Librado and Rozas 2009). To compare synonymous and nonsynonymous variation within and between species with the McDonald–Kreitman test, we used reduced sequence data sets of allopatric house sparrows and Spanish sparrows. Italian sparrows could generally not be tested against house sparrows due to their very similar mitochondrial gene pools (the contingency table could not be computed) and were excluded from this analysis.

## Results

### Habitat choice and breeding phenology

**Figure 2:**
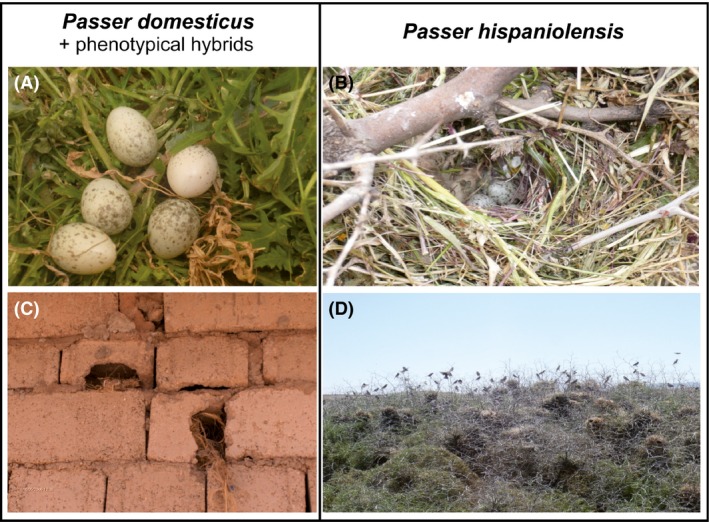
Nesting sites of house sparrows, Spanish sparrows in Algeria, and phenotypical hybrids; (A, C) *P. domesticus*: nest with eggs and burrows in brick wall, both at Djelfa – phenotypical hybrids show the same nesting site preference; (B, D) *P. hispaniolensis*: nest with eggs and breeding colony in jujube bushes, both at Hassi El‐Euch (photos: A. Ait Belkacem).

We surveyed a total of 103 nests in the Djelfa population (58 nests in 2011 and 45 nests in 2012), whereas, in Hassi‐El Euch, we surveyed 74 nests in the urban area (43 nests in 2011 and 31 nests in 2012) and 251 nests in the rural population (124 nests in 2011 and 127 nests in 2012). The two urban Algerian populations of Djelfa and Hassi El‐Euch were constituted by house sparrows, and phenotypical hybrid sparrows were exclusively associated with that species (“mixed urban populations” hereafter). In contrast, Spanish sparrows occurred only in the rural agricultural areas in the surroundings of the human settlements. In the rural surroundings of Djelfa, where jujube bushes were completely absent, no breeding Spanish sparrows were recorded during the two field seasons. In contrast, large breeding colonies existed throughout the Hassi‐El Euch area despite the low abundance of jujube bushes (<2% of the rural plots). In the urban areas, the great majority of house sparrow and phenotypical hybrid nests were located in holes of building walls (Fig. 2A and C; at Djelfa 90% of all nests in 2011 and 87% in 2012). All other nests were found in the vicinity or at human buildings, such as power poles. In contrast, all nests of the Spanish sparrows were built in colonies in large jujube bushes (*Zyziphus lotus*) along large wheat (*Triticum durum*) fields (Fig. 2B and D).

**Figure 3:**
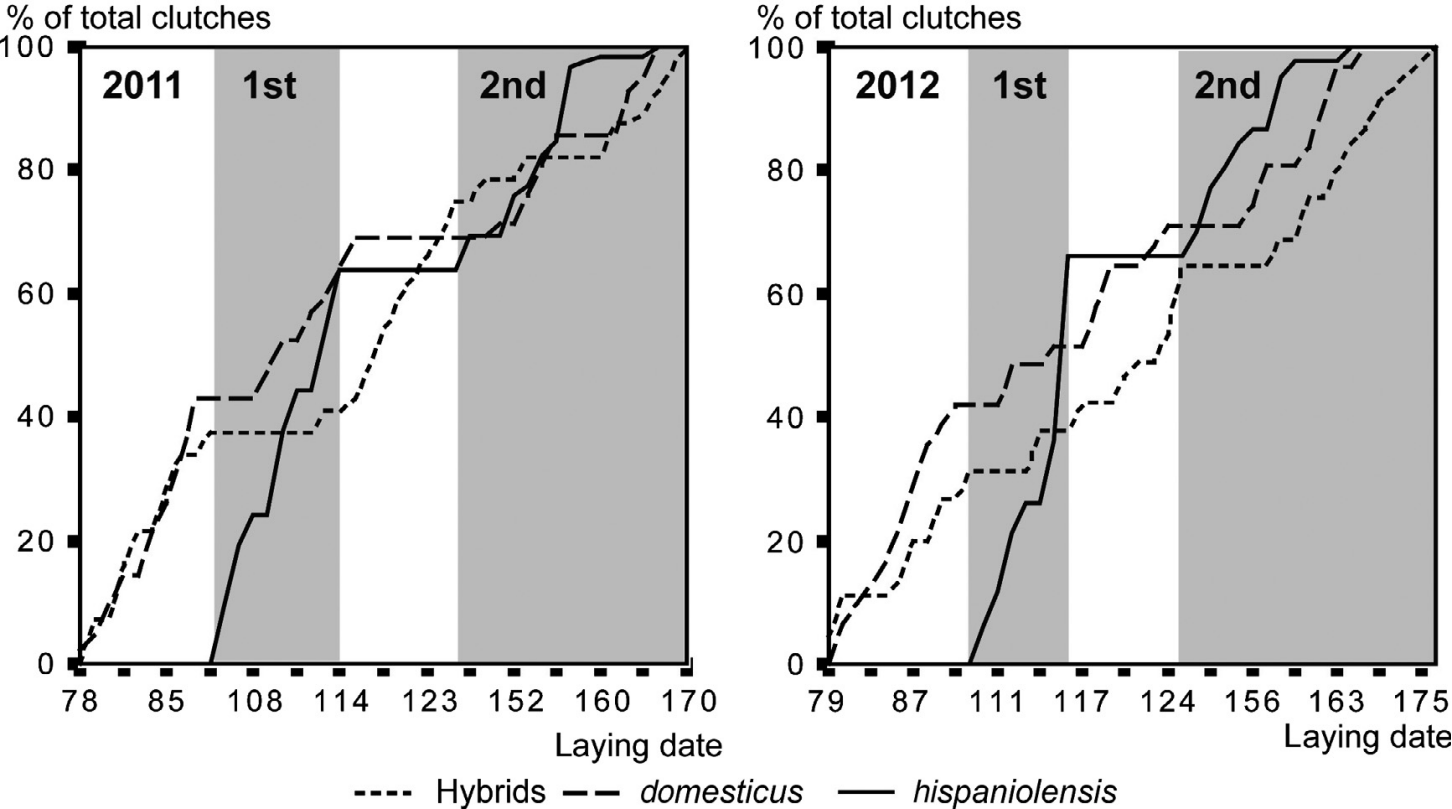
Breeding phenology of Algerian house sparrows (*P. domesticus*), Spanish sparrows (*P. hispaniolensis*), and their hybrids in the two study seasons; laying dates are numbered such that day 78 is March 19th and so on; *y*‐axis: percentage of total clutches of a species per season; laying periods of 1st and 2nd clutches of Spanish sparrows marked with gray shade; plateaus (with no increase of clutches initiated) indicate periods of incubation and parental care; note the synchronization of house sparrows' and Spanish sparrows' incubation and laying periods toward the end of the breeding season 2011 (less synchronized in the following year).

Breeding dates were similar between years (*F* = 1.575, *P* = 0.21), but consistently differed between populations (*F* = 5.523, *P* = 0.004) in the two study years (*F* = 0.329, *P* = 0.719). Post hoc analyses showed that the breeding dates were similar between the two mixed urban populations (*t* = −1.532, *P* = 0.273) and thus were simplified as a unique “population.” After that, Tukey's test confirmed that the *P. hispaniolensis* population breeds later than *P. domesticus* (*t* = 2.94, *P* = 0.003). Analyzed by clutches, *P. hispaniolensis* started the first clutch later than the two mixed urban populations by mid of April (at Djelfa: *t* = 43.99, *P* < 0.001; Hassi El‐Euch: *t* = 41.21, *P* < 0.001) and both mixed populations had similar breeding dates with first clutches laid during second half of March (*t* = 0.49, *P* = 0.87). In the second clutch, the mixed urban population at Djelfa bred significantly earlier than the mixed population at Hassi El‐Euch (*t* = −4.97, *P* < 0.001) and both of them, significantly earlier than the *P. hispanonlensis* population (Djelfa: *t* = −44.742, *P* < 0.001; Hassi El‐Euch: *t* = −42.516, *P* < 0.001). Breeding dates in the third clutch were similar for the two mixed urban populations (*t* = −1.404, *P* = 0.166). *P. hispaniolensis* did not lay a third clutch, and thus, their breeding period was shorter than that of neighboring house and hybrid sparrow populations. Breeding phenology of the two species and their hybrids was not entirely synchronized, because in both years laying periods of Spanish sparrows often coincided with incubation and parental care of house sparrows and hybrids (Fig. 3). Laying periods of the two species coincided most closely at the end of the breeding season, thus during second clutches of Spanish sparrows and third clutches of house sparrows and hybrids (Fig. 3).

Overall, clutch sizes and number of hatched chicks, which were unaffected by the breeding date (GLMM: *Z* = −0.056, *P* = 0.57 and *Z* = −0.504, *P* = 0.614, for egg and hatched chicks, respectively), were similar in the Spanish sparrow population and the two mixed urban populations (Table 1), which was confirmed in the analyses by clutch (all comparisons for first, second, and third clutches: *P* > 0.11).

**Table 1:** Results of nest surveys from a 2‐year period in two urban and one rural sparrow populations in Algeria (means for clutch size, laying and hatching dates, incubation time, number of hatched chicks (= Young), and breeding success ±SD)

	Year	Clutch no (*n*)	Laying (day)	Hatching (day)	Incubation (days)	Eggs (*n*)	Young (*n*)	Success (%)
*P. domesticus *+ hybrids Djelfa, urban	2011	1 (21)	83.7 ± 2.9	96.7 ± 2.9	13.0 ± 0.0	4.0 ± 0.8	3.4 ± 0.8	85.4 ± 17.2
		2 (18)	119.4 ± 3.5	132.8 ± 3.9	13.3 ± 0.5	3.5 ± 0.8	3.2 ± 0.9	91.0 ± 17.6
		3 (19)	158.1 ± 8.1	171.3 ± 8.1	13.3 ± 1.0	3.8 ± 0.9	3.1 ± 0.9	82.7 ± 20.5
	Total	*n* = 58				3.8 ± 0.8	3.2 ± 0.8	86.2 ± 18
	2012	1 (14)	86.0 ± 4.6	99.3 ± 4.8	13.3 ± 4.7	3.9 ± 0.7	3.4 ± 0.6	86.9 ± 16.5
		2 (15)	120.7 ± 4.5	134.1 ± 4.8	13.5 ± 0.6	3.9 ± 0.6	3.4 ± 0.5	84.8 ± 19.9
		3 (16)	166.1 ± 5.8	179.8 ± 5.4	13.7 ± 0.6	3.9 ± 0.8	3.3 ± 0.9	84.1 ± 17.9
	Total	*n* = 45				3.9 ± 0.7	3.3 ± 0.7	85.2 ± 18
*P. domesticus *+ hybrids Hassi El‐Euch, urban	2011	1 (18)	84.4 ± 3.1	97.3 ± 3.1	12.9 ± 0.2	4.1 ± 0.8	3.6 ± 0.7	88.7 ± 12.0
		2 (11)	112.1 ± 2.3	125.1 ± 2.5	13.0 ± 0.4	3.7 ± 0.8	3.3 ± 1.0	87.6 ± 21.0
		3 (14)	157.9 ± 4.9	171.9 ± 4.8	14.0 ± 0.4	3.9 ± 0.6	3.3 ± 0.6	86.5 ± 16.8
	Total	*n* = 43				4.0 ± 0.7	3.4 ± 0.8	87.7 ± 16
	2012	1 (13)	85.9 ± 2.6	97.9 ± 2.3	12.0 ± 0.6	4.1 ± 0.5	3.5 ± 0.7	86.2 ± 17.5
		2 (9)	117.7 ± 4.1	130.9 ± 4.2	13.2 ± 0.4	3.7 ± 0.7	3.0 ± 0.5	83.5 ± 16.4
		3 (9)	161.1 ± 3.3	174.9 ± 3.5	13.8 ± 0.4	3.8 ± 0.4	3.4 ± 0.7	93.5 ± 13.0
	Total	*n* = 31				3.9 ± 0.7	3.4 ± 0.7	87.6 ± 16
*P. hispaniolensis* Hassi El‐Euch, rural	2011	1 (79)	110.0 ± 2.9	123.0 ± 2.9	13.0 ± 0.0	4.0 ± 0.8	3.2 ± 0.7	80.5 ± 16.0
		2 (45)	154.4 ± 3.9	168.2 ± 4.0	13.8 ± 0.4	4.0 ± 0.9	3.4 ± 0.7	84.5 ± 14.5
	Total	*n* = 124				4.0 ± 0.8	3.2 ± 0.8	82.0 ± 16
	2012	1 (84)	114.0 ± 2.4	127.8 ± 2.5	13.8 ± 0.4	3.9 ± 0.7	3.1 ± 0.7	81.2 ± 16.4
		2 (43)	156.2 ± 3.8	169.7 ± 4.3	13.5 ± 0.7	3.9 ± 0.8	3.2 ± 0.8	83.1 ± 16.8
	Total	*n* = 127				3.9 ± 0.8	3.2 ± 0.7	81.8 ± 17

### Morphometry

**Figure 4:**
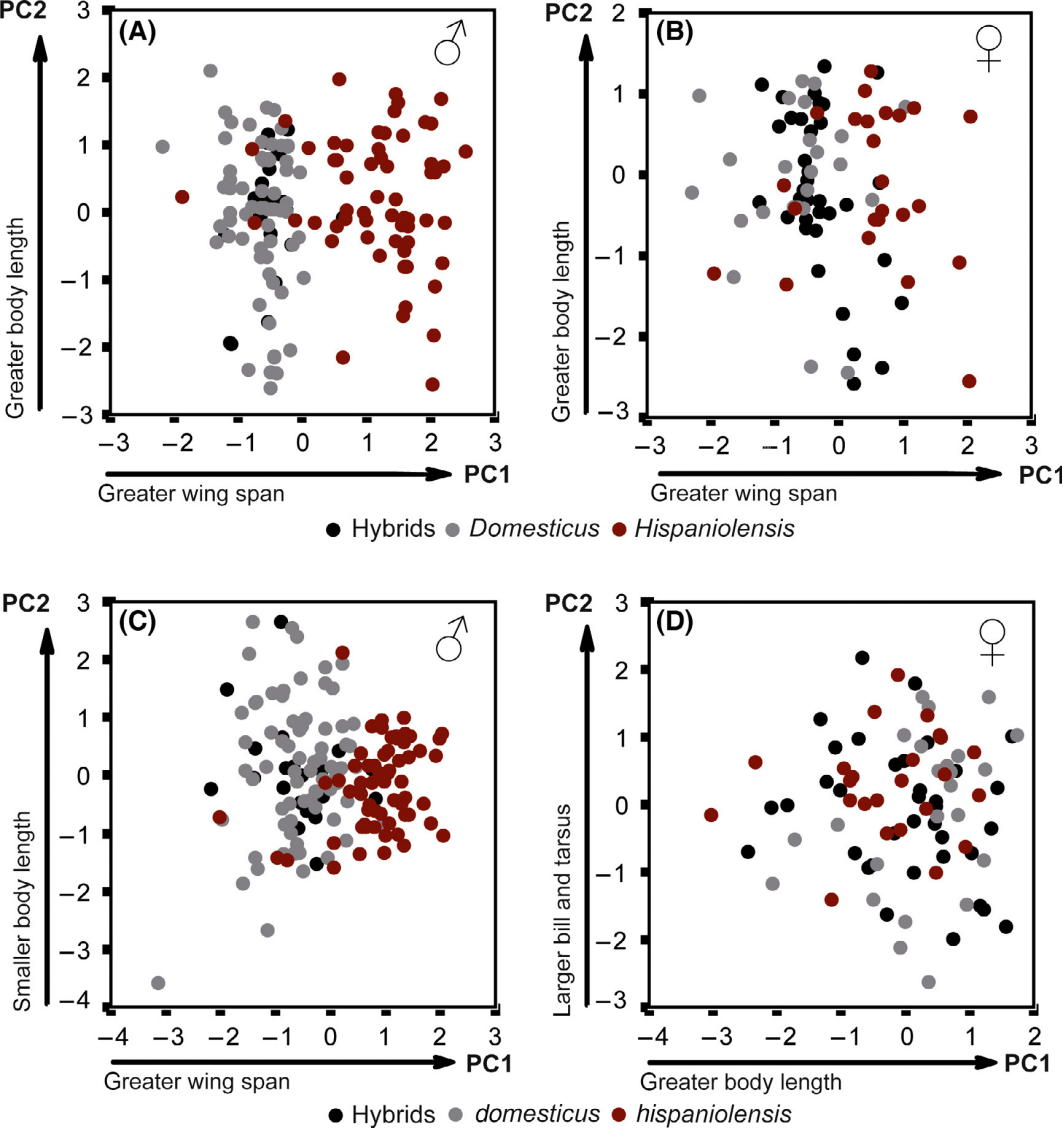
Morphometric variation among North African sparrows and their phenotypical hybrids in males (A) covariance matrix, (C) correlation matrix and females, (B) covariance matrix, (D) correlation matrix. Results are from principal component analysis (PCA) of four morphological parameters (body length, wing span, beak length, and tarsus length)

In both PCAs (covariance and correlation matrix approach), two separate clusters resulted from PCA with the data subset for males that corresponded to the two parental species (Fig. 4A). The first two principal components based on a correlation matrix explained a smaller percentage of the total variation (61.7%) but had slightly higher eigenvalues (1.43, 1.05) compared to the covariance matrix approach (98.2%, eigenvalue PC1 = 1.17, PC2 = 0.17; Table S1). Factor loadings were similar in the two analyses for males and females: PC1 was most heavily loaded by wing span, and PC2 was most heavily loaded by body length (except for PCA with females based on the correlation matrix, compare Table S1). In the scatterplots of PC1 vs. PC2, male phenotypical house sparrows and Spanish sparrows represented two largely separate clusters. Most *P. hispaniolensis* had positive values of PC1, whereas most *P. domesticus* had negative values and male phenotypical hybrids had an almost 100% overlap with the house sparrow cluster (Fig. 4A and C). The dimension of PC1 reflected that on average Spanish sparrows had a greater wing span compared to house sparrows and hybrids (Table 2), and unlike for all other biometric variables, the interaction phenotype:sex was significant for wing span (*F* = 7.61, *P* < 0.001). The separation of the two parental species was less well reflected by PCA results from the female data subset (Fig. 4B and D), but differences between the parental and the hybrid phenotypes in scores of PC1 and PC2 were significant for both sexes (for males: *F* = 166.58, *P* < 0.001; for females: *F* = 16.47, *P* < 0.001). Spanish sparrows differed significantly from house sparrows and hybrids (post hoc comparisons, Tukey's test, *P* < 0.001), whereas the latter two did not differ neither in males nor in females (Tukey's test, *P* = 0.71 for males and *P* = 0.13 for females).

**Table 2:** Means (±SD) of body size parameters for Algerian house sparrows (*P*. *domesticus*), Spanish sparrows (*P*. *hispaniolensis*) and their hybrids

	*N*	Weight (g)	Body length (cm)	Wing (cm)	Beak (cm)	Tarsus (cm)
*P*. *hispaniolensis*						
Male	63	26.7 ± 1.0	15.0 ± 0.4	25.3 ± 0.8	1.16 ± 0.04	3.27 ± 0.12
Female	24	25.7 ± 1.0	14.8 ± 0.4	24.6 ± 1.0	1.17 ± 0.04	3.13 ± 0.12
*P*. *domesticus*						
Male	71	26.3 ± 0.8	14.8 ± 0.4	23.4 ± 0.4	1.19 ± 0.06	3.21 ± 0.16
Female	24	25.7 ± 0.7	14.7 ± 0.4	23.4 ± 0.8	1.17 ± 0.06	3.13 ± 0.14
Hybrids						
Male	25	26.2 ± 0.8	14.8 ± 0.4	23.5 ± 0.4	1.19 ± 0.05	3.20 ± 0.12
Female	38	25.1 ± 0.6	14.7 ± 0.4	23.8 ± 0.5	1.17 ± 0.04	3.11 ± 0.16

### Mitochondrial DNA

**Figure 5:**
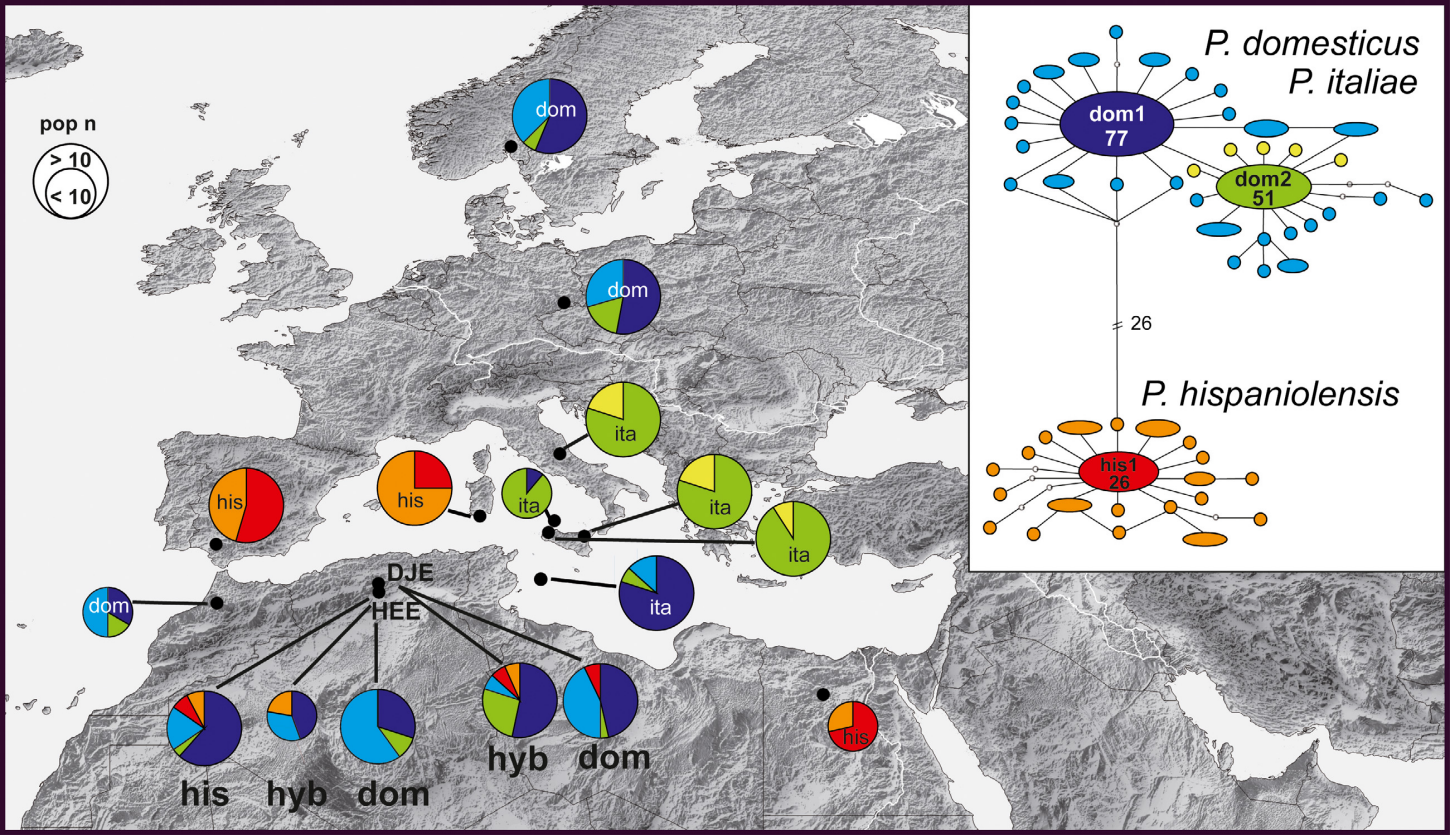
Distribution and frequency of ND2 haplotypes in European and Algerian study populations of house sparrows (dom), Spanish sparrows (his), Italian sparrows (ita), and North African hybrids (hyb). The haplotype network based on a 707‐bp fragment of the mitochondrial ND2 gene shows *P*. *domesticus* and *P*. *hispaniolensis* as two genetic clusters, separated by a minimum of 27 substitutions. Numbers in the network indicate the numbers of individuals sharing the three most common haplotypes. Pie charts show the frequency distribution of haplotypes for each population (according to color code of the network); Algerian study populations: DJE = Djelfa, HEE = Hassi El‐Euch.

The haplotype network was divided into two clusters separated by a minimum of 27 substitutions (Fig. 5). Most tip haplotypes differed by only one substitution from the most common haplotype of each cluster. The house sparrow cluster comprised 35 *P*. *domesticus* haplotypes, the most common central haplotype dom1 (*n* = 77 individuals) was found in all house sparrow populations and was also highly abundant in Algerian populations of Spanish sparrows and mixed urban populations. However, haplotype dom1 was nearly absent from *P. italiae* populations from the Italian Peninsula, Sicily and Ustica, whereas haplotype dom2 was the most abundant one there (Figs 5, S3). All other Italian haplotypes were unique variants derived from dom2 and did not occur in other study populations (Fig. 5). Despite being phenotypically close to Spanish sparrows, all birds from Lampedusa carried *P. domesticus* haplotypes, too. However, unlike in other *P. italiae* populations haplotype dom1 was the most abundant on Lampedusa island and dom2 was the rare variant there (Fig. 5). The Spanish sparrow cluster included 21 *P. hispaniolensis* haplotypes: the most common central one his1 (*n* = 26 individuals) was found in all Spanish sparrow populations, and at low abundances, it was also present in Algerian mixed urban populations (Figs 5, S1).

**Figure 6:**
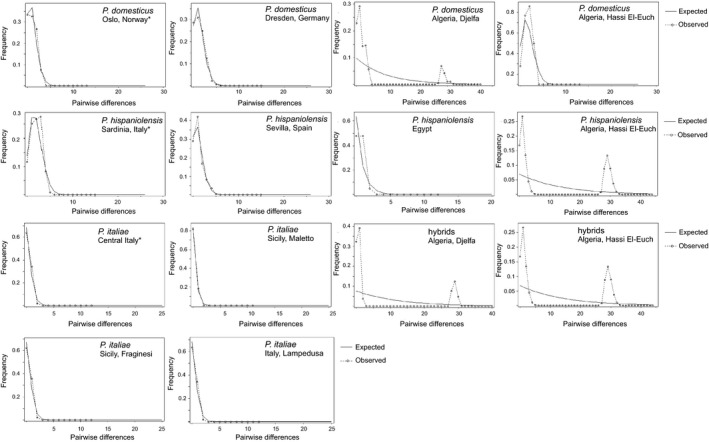
Observed (dash line) and expected (solid line) mismatch distributions for European and Algerian sparrow populations; *x*‐axis = pairwise number of differences, *y* = frequency; *haplotype data set from Elgvin et al. (2011).

MtDNA profiles of European sparrow populations (except Italy) were in 100% accordance with the local phenotype: House sparrow populations included only *P. domesticus* haplotypes, whereas in Spanish sparrow populations, only *P. hispaniolensis* haplotypes were found (Fig. 5). Accordingly, mismatch distributions for these four populations (Fig. 6) were unimodal and diversity indices were relatively low (Table 3; with lowest values for haplotype and nucleotide diversity in Spain (*P. hispaniolensis*) and Norway (*P. domesticus*). The same was true for the limited samplings from Morocco (house sparrows only) and from Egypt (Spanish sparrows only; Figs 5, 6, Table 3). Unimodal mismatch distributions of Italian peninsular and island populations of *P. italiae* were extremely steep due to very low intraspecific genetic diversity. This was confirmed by low values for haplotype diversity (h) and nucleotide diversity (*π*) of Italian populations (0.18 < h < 0.38; 0.00026 < *π *< 0.00057) compared to much higher values for all house sparrow and Spanish sparrow populations (0.52 < h < 0.93; 0.0081 < *π *< 0.003; Table 3). Among all study populations, only house sparrows of Sevilla showed a significantly negative Tajima's D (Table 3). McDonald–Kreitman test with the parental species data set, however, did not confirm a general deviation from neutrality for the ND2 fragment analyzed: 22 synonymous substitutions vs. 1 nonsynonymous substitution at 31 polymorphic sites, neutrality index, NI (Jukes–Cantor) = 0.714, *P* = 0.696.

**Table 3:** Genetic diversity for European and Algerian populations of house sparrows (*P*. *domesticus*), Spanish sparrows (*P*. *hispaniolensis*) and their hybrids; local mixed Algerian populations included *domesticus* and hybrids but not *P*. *hispaniolensis*

Taxon	Population	*n*	h	hd	*π*	TD
*P. hispaniolensis*	Sevilla	22	10	0.710 ± 0.106	0.00165 ± 0.00042	−2.12492*
	Sardinia	16	7	0.883 ± 0.045	0.00281 ± 0.00043	−0.63940
	Hassi El‐Euch	26	9	0.625 ± 0.109	0.01565 ± 0.00370	0.64309
	Egypt	7	3	0.524 ± 0.209	0.00081 ± 0.00036	−1.23716
*P. domesticus*	Dresden	17	7	0.713 ± 0.109	0.00185 ± 0.00046	−1.58056
	Oslo	16	5	0.667 ± 0.113	0.00153 ± 0.00034	−0.31696
	Hassi El‐Euch	10	7	0.911 ± 0.077	0.00239 ± 0.00039	−0.84270
	Djelfa	28	11	0.772 ± 0.077	0.00733 ± 0.00324	−1.56949
	Morocco	6	5	0.933 ± 0.122	0.00312 ± 0.00102	−0.93169
Phenotypical hybrids	Hassi El‐Euch	9	6	0.833 ± 0.016	0.01684 ± 0.00668	0.04907
	Djelfa	15	5	0.676 ± 0.105	0.01079 ± 0.00524	−0.84678
*P. italiae*	C Italy	15	3	0.362 ± 0.145	0.00054 ± 0.00023	−1.00161
	Fraginesi	10	3	0.378 ± 0.181	0.00057 ± 0.00029	−1.40085
	Maletto	11	2	0.182 ± 0.144	0.00026 ± 0.00020	−1.12850
	Ustica	9	2	0.222 ± 0.166	0.00031 ± 0.00024	−1.08823
	Lampedusa	15	3	0.362 ± 0.145	0.00054 ± 0.00023	−1.00161

*N*, sample size; h, number of haplotypes; hd, haplotype diversity ± SD; *π*, nucleotide diversity ± SD; TD, Tajima's D (*Significance level *P* < 0.05).

Unlike European populations, almost all Algerian populations had a bimodal mismatch distribution due to the local co‐occurrence of both *P. domesticus* and *P. hispaniolensis* haplotypes (Fig. 6). Their mtDNA profiles were not in accordance with the existing phenotypes, except for the phenotypical house sparrows from Hassi El‐Euch city that exclusively carried *P. domesticus* haplotypes (Fig. 5). Generally, all Algerian populations were admixed, but house sparrow mtDNA was locally most abundant regardless of phenotypical variation (Figs 5, 6). For example, 75% of the rural, phenotypical Spanish sparrows at Hassi El‐Euch carried a *P. domesticus* haplotype. Likewise, most house sparrows and phenotypical hybrids in urban populations carried a *P. domesticus* haplotype, but a low percentage of individuals at Djelfa carried the most common *P. hispaniolensis* haplotype. Due to strong mtDNA admixture, Algerian populations did not greatly differ in haplotype diversity from European and other North African populations, but had eight to ten times higher nucleotide diversity (Table 3).

## Discussion

While there is increasing information available on the ecological and genetic differentiation of the Italian hybrid form, *P. italiae,* from the two parental species (Hermansen et al. 2011, 2014; Elgvin et al. 2011; Tu 2012; Trier et al. 2014), comparable knowledge from North Africa is limited. In this context, we document substantial differences between European and Algerian populations for the first time with respect to (1) the local spatial distribution of the three forms and (2) the genetic constitution of populations. Field observations on the species' ecological preferences and breeding biology might help explaining these differences.

### Habitat choice

In our Algerian study populations, house sparrows bred in urban areas, whereas Spanish sparrows tended to avoid human settlements and were strongly restricted to cultivated areas. These different ecological preferences of the two sparrow species in local sympatry are in good accordance with the situation in other parts of North Africa (Meise 1936; Summers‐Smith and Vernon 1972; Riss 1989) and on the Iberian Peninsula (Alonso 1986; Murgui 2011; Tu 2012). Generally, ecological segregation of the two sparrow species might be more depending on nest site choice than on adaptation to different food resources, because dietary preferences of house sparrows and Spanish sparrows largely overlap on the European continent (Summers‐Smith 1988; Bernis 1989; Cramp and Perrins 1994; Haffer and Hudde 1997; Cordero 2004) and in North African populations (El Kharrim et al. 1997; Belkacem et al. 2012). Differences in bill dimensions that may indicate adaptation to different food niches were not found in our Algerian study populations. However, bill size dimensions in sparrows are strongly influenced by abiotic factors (e.g., precipitation regimes) and, apparently, the degree of parental genetic contribution does not affect the large phenotypic variation of bills (Eroukhmanoff et al. 2013).

In accordance with previous records from Algeria and Tunisia (Summers‐Smith and Vernon 1972), hybrid individuals were always found in urban habitats, associated with house sparrows (*P. domesticus*) and like the latter they occupied nesting holes in human buildings. It seems that habitat choice of North African hybrids strongly depends on the presence of either of the parental species, because in the absence of Spanish sparrows, large hybrid sparrow populations were found in oases agrosystems such as date palm groves, but the preference for nest sites on human buildings remained unaltered in these hybrid colonies (Guezoul et al. 2010, 2011; Guezhoul et al. 2013). Shared habitat preferences between hybrids and one of the parental species have also been reported in another well‐studied hybrid zone of great tits and Japanese tits (*Parus major*,* P. minor*) in Far East (Kvist et al. 2003, Kvist and Rytkönen 2006). There, the western species, *P. major,* and the similar‐sized hybrids occupy the towns whereas the smaller Asian, *P. minor,* breeds outside the human settlements (Nazarenko et al. 1999; Päckert et al. 2005; Fedorov et al. 2009). Strikingly, the great tit and the house sparrow are synanthropic species that are well adapted to human settlements. Their radiation and range expansion were strongly associated with man‐made structures (a recent eastward expansion of great tits along the Trans‐Siberian Railway; Kapitonova et al. 2011) or with human commensalism in the sparrows (Sætre et al. 2012). Like in the great tit example, hybridization among the two sparrow species in North Africa is very likely a quite recent process. Before 1900, only rare and isolate records of hybrid individuals existed east of 2°E. However, in the first quarter of the 20th century, hybrids records steadily increased in frequency and extension range, likely as a result of the successive arrival of house sparrows to the cities (Summers‐Smith and Vernon 1972). As a consequence, the successful adaptation urban habitats by one species might have involved selective advantages in several life history traits compared to their rural counterparts (Møller 2009). Therefore, it seems that anthropogenic land‐use change may have promoted hybridization and hybrid range expansion in North African sparrows like it was suggested for the origin and rapid dispersal of the hybrid Italian sparrow (Hermansen et al. 2011).

### Breeding phenology

Compared to Algerian mixed populations of house sparrows and hybrids in the urban areas, Spanish sparrows bred later and raised only two clutches instead of three. In other Algerian populations of pure hybrids, breeding phenology was very similar to those from our urban study populations: Courtship began in early February and the first of three successive broods started in mid‐March (Guezoul et al. 2011). In local study populations on the Iberian Peninsula, the two parental sparrow species started first broods at almost identical dates, but house sparrows had more successive clutches, and thus longer breeding periods than Spanish sparrows (Alonso 1983). In contrast, in other populations, Spanish sparrows bred up to 13 days later than house sparrows (Tu 2012). The shorter breeding period of Spanish sparrows compared to that of house sparrows in Algerian and European populations might well be related to the migratory or nomadic behavior of the species on the European continent (Haffer and Hudde 1997 and references therein) and North Africa (Bachkiroff 1953; Bortoli 1973; Cramp and Perrins 1994; Haffer and Hudde 1997; White et al. 2013). Migratory behavior of Spanish sparrows may also explain the observed differences in body size parameters found between the two study species (i.e., greater wing span in *P. hispaniolensis* than *P. domesticus*), because long and pointed wings in relation to other morphometric parameters can indicate a greater migratoriness of populations (Pérez‐Tris and Telleria 2001; Baldwin et al. 2010; Nowakowski et al. 2014; Hahn et al. 2016; but see Mönkkönen 1995). It has been suggested that even minimal differences in the timing of breeding phenology may act as premating isolation mechanism and prevent hybridization between house sparrows and Spanish sparrows (Tu 2012). This explanation, however, seems unsatisfying for our study area, because the breeding periods of the two species greatly overlap and both have more than one clutch. Furthermore, although the breeding period of Spanish sparrows started about 1 month later than that of house sparrows, a considerable number of hybrids were present in our study populations and mtDNA introgression – although largely unidirectional – was apparent even in the two parental species. Thus, further explanations for such considerable degree of mitochondrial gene flow have to be considered.

### Molecular genetics – Asymmetric mitochondrial introgression in North Africa

Local compositions of mitochondrial gene pools suggest different demographic histories of sparrow hybrid populations on the Italian Peninsula and on the North African continent, respectively. While in *P. italiae* the house sparrow mitogenome is near‐fixed (Elgvin et al. 2011; Hermansen et al. 2011), a small percentage of Algerian hybrids carried *P. hispaniolensis* haplotypes. Furthermore, we observed strong mitochondrial introgression of house sparrow mitogenome into the North African *P. hispaniolensis* populations. The small percentage of *P. hispaniolensis* haplotypes in the urban house sparrow population at Djelfa shows that mitochondrial introgression is largely but not completely unidirectional in these North African populations. Thus, we would assume that despite spatial separation hybridization between the two sparrow species in Algeria is not a historical process, but still ongoing. The extent and the directionality of genetic introgression across a hybrid zone can be generally affected by complex interactions of sexual selection (Helbig et al. 2001; While et al. 2015) and natural selection, such as differential adaptation to local environments (Rheindt 2011; Walsh et al. 2016). Furthermore, there is mixed evidence that niche divergence itself might promote asymmetrical introgression among hybridizing species (Wielstra and Arntzen 2014; Jiménez and Ornelas 2016). Hermansen et al. (2011) argued that hybridization among house sparrows, Spanish sparrows, and their hybrids was limited to regions of low population densities such as the contact zone of house sparrows and Spanish sparrows in the Alps and in North Africa and the Cape Verde Islands, where one of the species is supposedly rare. For such numerically imbalanced populations, the desperation hypothesis (Hubbs 1955) predicts that restricted mate choice in the rare species should increase the possibility heterospecific matings and promote hybridization processes. Although empirical support for Hubb's principle has been found in a number of species, most of them dealt with single hybrid individuals (Beier et al. 1997; McCracken and Wilson 2011; Ralston et al. 2015), and thus, this theory does not serve as a general rule (for counterevidence see Randler 2008). In fact, field data from North Africa showed that none of the three sparrow phenotypes is actually rare (Guezoul et al. 2010, 2011; Bendjoudi et al. 2013), but nevertheless, the spatial separation among populations from urban and rural environments might enhance mixed matings in a way that house sparrows (and hybrids) are rare in rural Spanish sparrow populations and vice versa. Unfortunately, the actual percentage of mixed matings in North African sparrow populations has not been estimated yet and due to the problematic identification of female phenotypes that will remain a challenge to future field studies.

### Molecular genetics – Diversity loss and genetic drift in the Italian sparrow, *Passer italiae*

For the origin of the Italian hybrid form, *P. italiae*, Hermansen et al. (2011) developed a recent Holocene scenario and assumed hybridization processes to be strongly linked to the intensification of agriculture at about 10,000 years ago (compare Sætre et al. 2012). Undoubtedly, the mitochondrial gene pool of the house sparrow (including variants dom1 and dom2) must have diversified quite recently, but a late Pleistocene scenario for the origin of the Italian hybrid is a plausible alternative for some reasons. First, the Italian Peninsula is one of three classical Southern European glacial refugia (along with the Iberian Peninsula and the Balkan Peninsula; Schmitt 2007; Stewart et al. 2010) from where different genetic lineages of some bird species originated and expanded their ranges after glacial retreat (Brito 2005; Brambilla et al. 2008). In contrast to the flanking regions to the West and the East, it seems that the Italian Peninsula constituted a more restricted and isolated refugium (particularly when compared to the Balkans) because Italian genomes of phylogeographically structured species rarely populated Central and Northern Europe as the Alps acted as strong barrier to gene flow (Hewitt 2000). Statistical tests of selective neutrality did not indicate a strong signal of selection or population growth in populations from the classical refugia except for the Iberian population of *P. hispaniolensis*. However, in particular the ancestral *P. italiae* populations might have also been bottlenecked in their Italian refugium according to their extremely left‐skewed mismatch distributions and comparatively low genetic diversity. A similar loss of genetic diversity in Italian glacial refuges was confirmed for other terrestrial species (Deffontaine et al. 2005). Furthermore, although Elgvin et al. (2011) already found that Italian haplotypes do not form separate haplogroups, Italian populations differed from all our study populations in their genetic composition with respect to the high abundance of the elsewhere rare haplotype dom2 and the near absence of the elsewhere most abundant haplotype dom1. Such near‐fixation of rare haplotypes can also be a further possible effect of genetic drift in Pleistocene refugia (Bayard deVolo et al. 2013). Thus, the extant genetic diversity observed in *P. italiae* is likely the result of mitochondrial capture and near‐complete fixation of rare house sparrow haplotypes during past hybridization processes in glacial Italian refuges. Similar scenarios were developed for Mediterranean gulls (Liebers et al. 2004, 2010) and the Carpathian newt (Babik et al. 2005; Zieliński et al. 2014). The closer resemblance of the *P. italiae* population from Lampedusa (with dom1 being the most abundant haplotype) with *P. domesticus* and Algeria hybrid populations is likely due to the close proximity of the island to the African continent from where founder populations might have colonized the island. This underlines the complex phylogeographic pattern of Mediterranean hybrid sparrow populations, because despite their strong phenotypical similarity to Spanish sparrow populations from Sicily and Lampedusa, they greatly differ with respect to frequencies of the two most common house sparrow haplotypes.

## Conclusion

In the North African agricultural landscape mosaic, the patchy distribution of the two sparrow species and their hybrids is a unique spatial pattern that is not found elsewhere across the sympatric Eurasian range of house sparrows and Spanish sparrows and the isolated range of the Italian hybrid form. Despite different ecological preferences and timing of the breeding period, the North African populations are characterized by strongly imbalanced mitochondrial introgression of urban house sparrow haplotypes into the rural Spanish sparrow populations. Compared to North African phenotypical hybrid populations, local Italian gene pools of the stabilized hybrid *P. italiae* are characterized by low genetic diversity and high abundances of rare haplotypes. Such differences between mitochondrial gene pools of Italian and North African hybrids provide first evidence that different demographic histories have shaped the extant genetic diversity observed on both continents. Although mitochondrial genetic diversity and phylogeographic patterns have a limited explanatory power for the study of hybridization processes, it may be informative only for taxon pairs, like the two sparrow species, represented by distinct phenotypes. However, only nuclear markers allow for unmistakable identification of genetic hybrid individuals and populations and a more comprehensive reconstruction of evolutionary scenarios, historical demography, or colonization pathways as shown in sparrows (Elgvin et al. 2011; Hermansen et al. 2011, 2014; Trier et al. 2014). Whether strongly asymmetrical mitochondrial introgression among North African sparrow populations is reflected by patterns of nuclear gene flow will set a future challenge to follow‐up studies.

## Conflict of Interest

None declared.

## Supporting information


**Figure S1.** Phenotypical diagnosis of target sparrow species in Algerian populations; A) five major plumage traits that are distinctive for house sparrows (B: *P. domesticus*) and Spanish sparrows (C: *P. hispaniolensis*) but are intermediate to a variable degree in a considerable number of putative hybrid individuals (D) per local population (*P. domesticus* × *P. hispaniolensis*).

**Figure S2.** Phenotypical comparison of Mediterranean island populations (Sicily, Ustica, Lampedusa) of the Italian hybrid form, *P. italiae*.

**Figure S3.** Haplotype network of European and North African sparrow populations (*P. domesticus*,* P. hispaniolensis*,* P. italiae* and North African hybrids) based on 707 bp of the mitochondrial ND2; populations of origin are color‐coded for each haplotype.

**Table S1.** Results of principal component analysis (PCA) of four biometric measurements (length of body, wing, bill and tarsus) from male and female house sparrows (*P. domesticus*), Spanish sparrows (*P. hispaniolensis*) and their hybrids; eigenvalues and factor loadings for the first two principal components (PC1, PC2) based on a covariance matrix and based on a correlation matrix; % = percentage of the total variation explained by one component.
