# Supplementary figures and images for: North African hybrid sparrows (Passer domesticus, P. hispaniolensis) back from oblivion – ecological segregation and asymmetric mitochondrial introgression between parental species
Source: Ecol Evol. 2016 Jun 28;6(15):5190–206. doi: 10.1002/ece3.2274 (PMC4984497; doi:10.1002/ece3.2274)

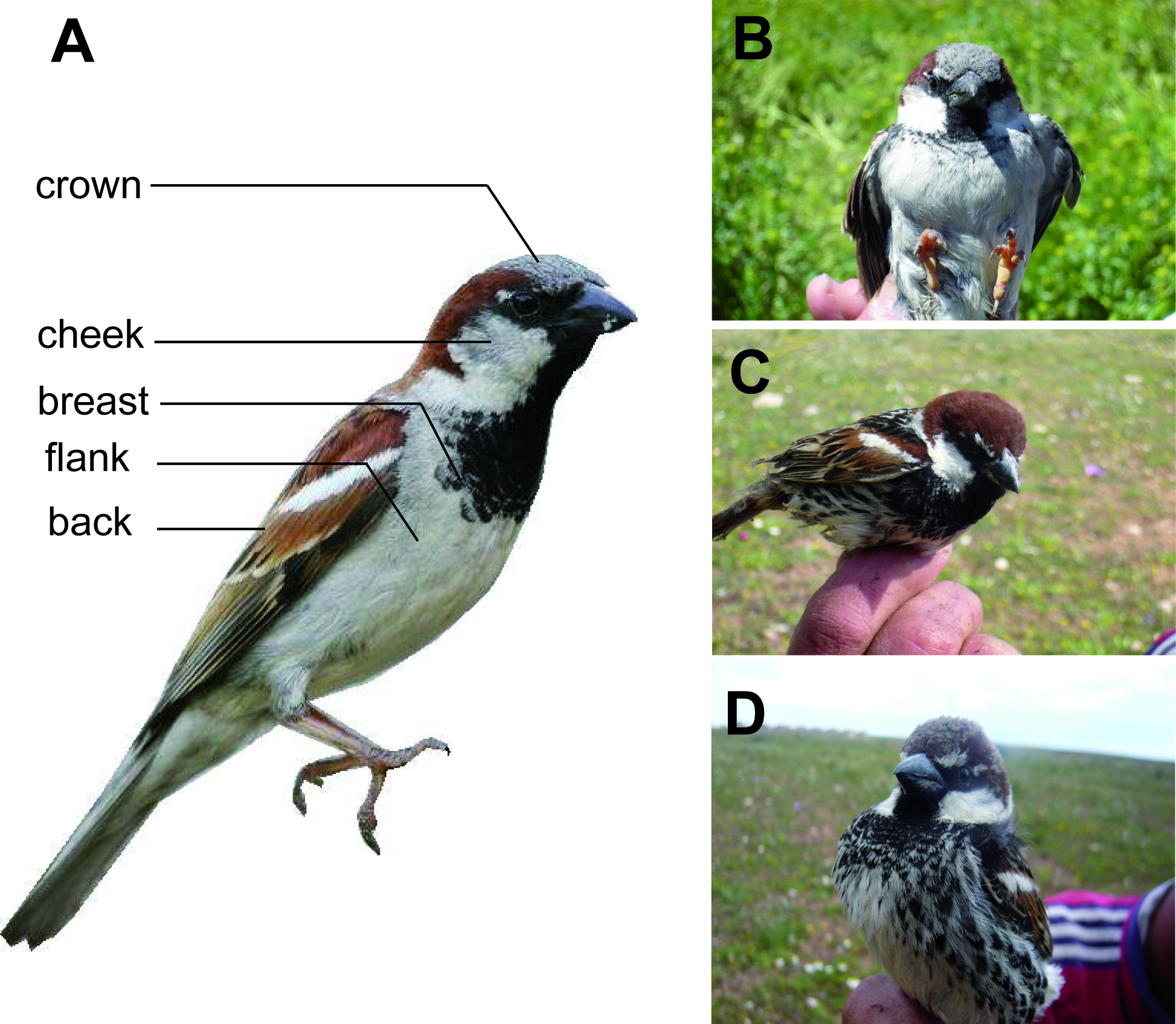

Supplement: Supplementary file 1 — Figure S1. Phenotypical diagnosis of target sparrow species in Algerian populations; A) five major plumage traits that are distinctive for house sparrows (B: P. domesticus) and Spanish sparrows (C: P. hispaniolensis) but are intermediate to a variable degree in a considerable number of putative hybrid individuals (D) per local population (P. domesticus × P. hispaniolensis). [file ECE3-6-5190-s001.jpg]

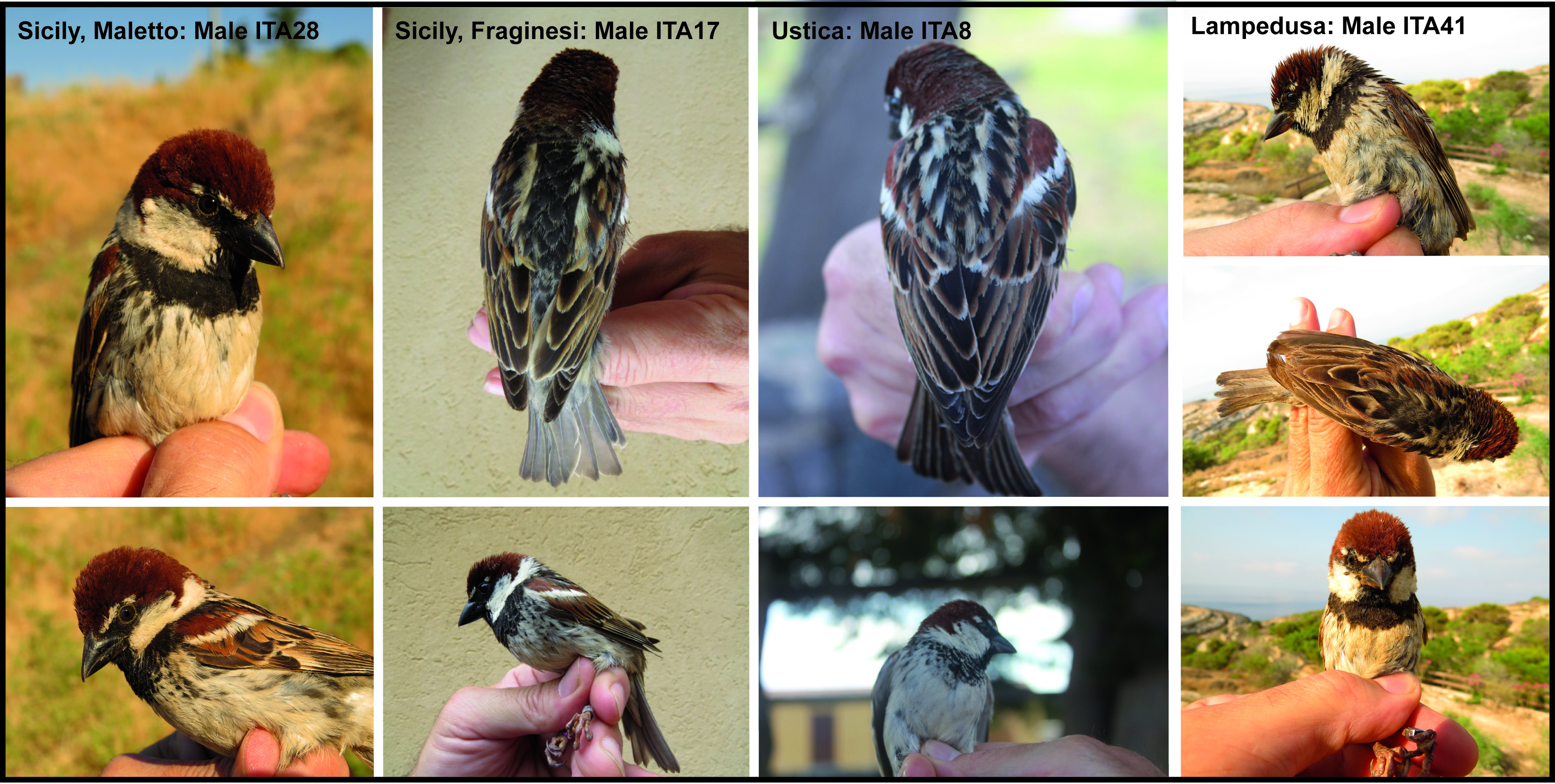

Supplement: Supplementary file 2 — Figure S2. Phenotypical comparison of Mediterranean island populations (Sicily, Ustica, Lampedusa) of the Italian hybrid form, P. italiae. [file ECE3-6-5190-s002.jpg]

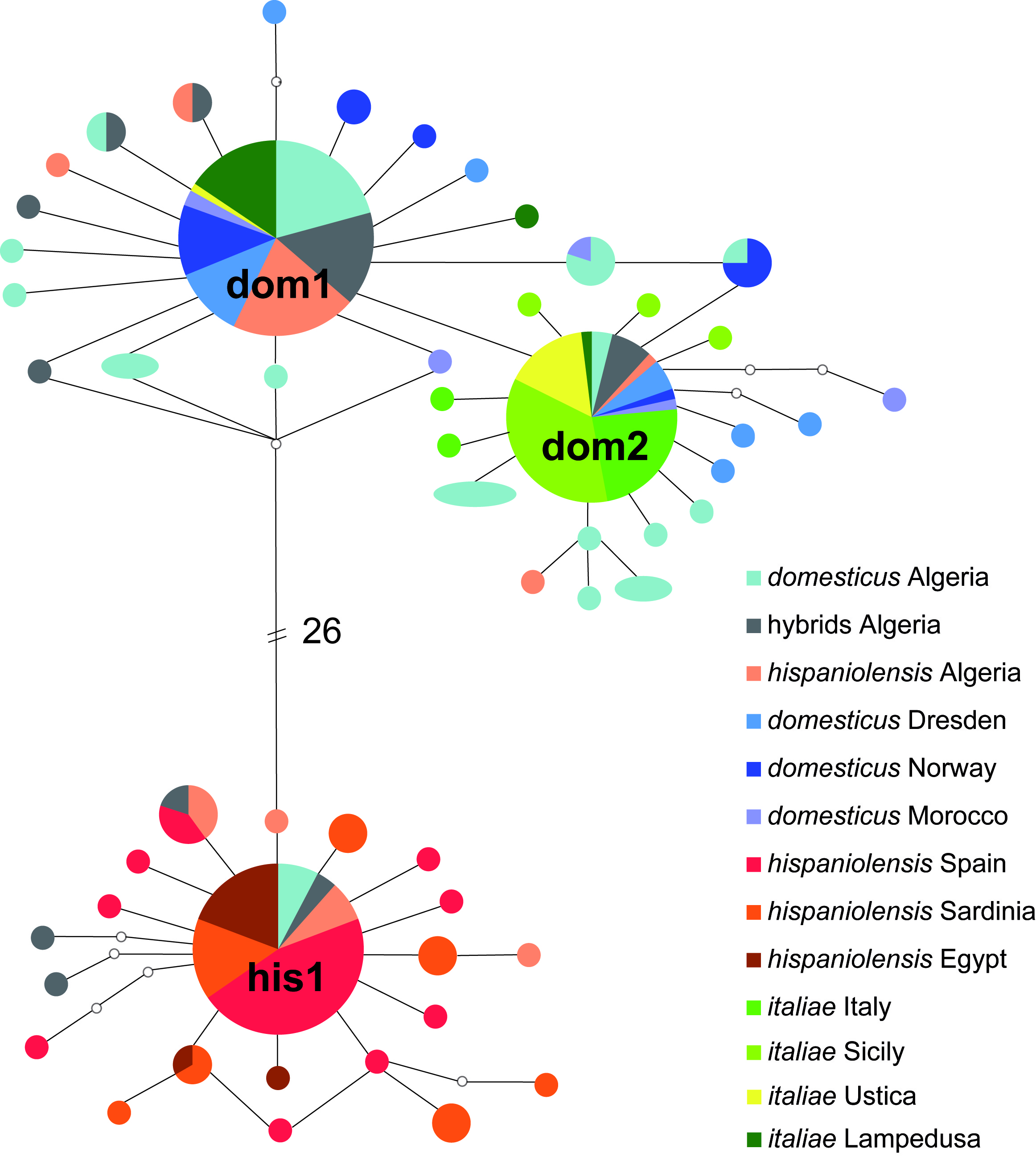

Supplement: Supplementary file 3 — Figure S3. Haplotype network of European and North African sparrow populations (P. domesticus, P. hispaniolensis, P. italiae and North African hybrids) based on 707 bp of the mitochondrial ND2; populations of origin are color‐coded for each haplotype. [file ECE3-6-5190-s003.jpg]
