# Supplementary material for: North African hybrid sparrows (Passer domesticus, P. hispaniolensis) back from oblivion – ecological segregation and asymmetric mitochondrial introgression between parental species
Source: Ecol Evol. 2016 Jun 28;6(15):5190–206. doi: 10.1002/ece3.2274 (PMC4984497; doi:10.1002/ece3.2274)
Supplement: Supplementary file 4 — Table S1. Results of principal component analysis (PCA) of four biometric measurements (length of body, wing, bill and tarsus) from male and female house sparrows (P. domesticus), Spanish sparrows (P. hispaniolensis) and their hybrids; eigenvalues and factor loadings for the first two principal components (PC1, PC2) based on a covariance matrix and based on a correlation matrix; % = percentage of the total variation explained by one component. [file ECE3-6-5190-s004.docx]

Table S1: Results of principal component analysis (PCA) of four biometric measurements (length of body, wing, bill and tarsus) from male and female house sparrows (*P. domesticus*), Spanish sparrows (*P. hispaniolensis*) and their hybrids; eigenvalues and factor loadings for the first two principal components (PC1, PC2) based on a covariance matrix and based on a correlation matrix; %= percentage of the total variation explained by one component.

| **males** | covariance | correlation | covariance | correlation |
| --- | --- | --- | --- | --- |
| **n=** | PC1 | PC1 | PC2 | PC2 |
| **eigenvalue** | 1.17 | 1.43 | 0.17 | 1.05 |
| **%** | 85.5 | 35.6 | 12.7 | 26.1 |
| **body** | 0.069 | 0.248 | 0.415 | -0.760 |
| **wing** | 1.079 | 0.845 | -0.026 | -0.004 |
| **bill** | -0.012 | -0.472 | 0.000 | 0.382 |
| **tarsus** | 0.047 | 0.654 | -0.015 | 0.568 |
|  |  |  |  |  |
| **females** | covariance | correlation | covariance | correlation |
| **n=** | PC1 | PC1 | PC2 | PC2 |
| **eigenvalue** | 0.79 | 1.22 | 0.16 | 1.15 |
| **%** | 81.5 | 30.6 | 16.2 | 28.7 |
| **body** | -0.024 | 0.771 | 0.395 | 0.152 |
| **wing** | 0.887 | -0.445 | 0.011 | 0.399 |
| **bill** | -0.001 | 0.516 | 0.009 | 0.676 |
| **tarsus** | 0.017 | -0.404 | -0.019 | 0.713 |
